# Report of Cases of Typhus Fever, Observed at the Lazaretto, near Philadelphia

**Published:** 1847-11

**Authors:** 


***Report of Cases of Typhus Fever, observed t the Lazaretto, near Philadelphia.*** By F. W. Sergent, M. D.

During the month of June, I had the opportunity of observing, at the Quarantine Station, several cases of "Ship Fever," as it is called. The following notice has been condensed from observations made at the time.

There were thirty-seven cases of fever in all, of which thirty-three were taken from one ship, "The North Star;" the remaining four were takon from two other vessels, two from each. The two latter ships sailed from Belfast and Londonderry respectively; having on board their full complement of passengers, generally in good condition, and pretty well furnished with provisions and other necessaries. The "North Star" sailed from Liverpool with one hundred steerage passengers. Very many of them excessively poor, and already suffering in health in consequence of the discom forts to which they had been subjected in Ireland, and also in Liverpool, whilst waiting for a passage. The bread-stuffs which were laid in for their consumption during the voyage, were of a very inferior quality.

The general impression on board the vessel, was, that one of the passengers, a woman, was sick when she embarked. This person was extremely poor, and had been compelled to remain in Liverpool many weeks, amidst a crowd of emigrants, in a very miserable condition. She remained sick from the time the vessel sailed, during the whole voyage, aud was removed from the ship at the Lazaretto, in a state of great prostration, yet free from fever.

The ship sailed from Liverpool on the 7th of May. The first death occurred on the 17th of May, in the case of one of the children of the woman above mentioned. On the 29th, another child of the same woman died. On the 13th of June her two remaining children were also removed; the ages of the four, in the order above mentioned, were 4 years, 9 mnoths, 2 years, and 5 years. In all, eight persons died on the voyage.

With regard to the question of contagion, sufficient data could not be gained to afford any satisfactory conclusion. It is important to note, however, that the captain of the ship, both mates, the cook and seven of the crew sickened, either during the voyage, or immediately on reaching the quarantine ground, and all presented well-marked symptoms, such as were offered by the sick passengers. These men were all healthy and vigorous, as well fed and as comfortably provided for, as seamen generally are. Moreover, Dr. Jones, the regularly constituted Lazaretto physician, contracted typhus fever, while in attendance at the station upon some fever patients taken from on board ship, in May last. No case of fever has occurred among the nurses of the station.

The symptoms presented by the patiants observed, were ascertained by careful and repeated inquiry of themselves and their friends, and by attentive observations at the bed-side. They may be divided into the symptoms of the disease in its forming stage, and those of the fully developed affection.

The disease was ushered in by chillness, in many of the cases, perhaps in all. The numerical frequency of this symptom could not of course be ascertained precisely, because many of the patients, when they were first brought under my notice, were not qualified to give information on this point, neither had their earliest complaints been observed by others. In some cases repeated rigors were experienced; in others the sensation was merely of chillness. Pain in the back and limbs was a frequent symptom, and in one patient, whom I had the opportunity of observing from the commencement of her illness, the pain in the sacral region was exceedingly severe, as much so as in very bad cases of small-pox;—this woman died. Without exception all the patients complained of headache; this was variable in intensity, sometimes very severe, as intimated on the part of the sufferers by the expressions "splitting," "bursting," &c., of the head; in other cases it was more supportable in its degree. The seat of the pain was not fixed; sometimes it affected the frontal, sometimes the superior, and sometimes the posterior region of the head. A marked degree of sleeplessness characterized the onset of the sickness in every case; in the instance of the captain of the ship, the want of sleep was so severely felt that he took every night, before reaching the quarantine ground, large quantities of laudanum; which, however, failed of its intended effect. In all the cases brought under my notice, delirium was a symptom present from an early period. Generally this was of a quiet, manageable character, the patients keeping their berths, and exhibiting their aberration only in random, unconnected talking, more or less obstinacy in refusing artention and assistance, aversion to their children or friends. Some, on the other hand, were with difficulty restrained from wandering about the ship, and from making a great deal of noise, &c. &c. The more active delirium was most marked at night. Loss of strength was also a notable phenomenon connected with this stage of the disorder: it was common as well among the previously robust and well-nourished sailors, as among the passengers. This prostration affected the mind as well as the body. The sick generally became utterly careless and indifferent as to their own situation and condition, and unsolicitous for their nearest friends. In other instances, where there was every disposition to the performance of accustomed duty, the mind seemed to have lost all power of observation, and combination of ideas, and was incapacitated, equally with the body, for exertion; thus the captain and the mates became entirely unable to calculate the position, or to lay down the course of the vessel.

More or less fever was observable almost synchronously with the first complaint, in those patients whom I saw at the beginning of their sickness. The pulse in the first period, beat from 90 to 100; soft, regular, of good volume—the skin was warm and moist; the tongue covered with a thin, moist yellowish fur, excepting at the anterior extremity, which was clean; the bowels were not disturbed at first; indeed, in all the cases, according to the most accurate information I could gain, the habit was costive at the commencement; the abdomen was in every case retracted, rather than full; nausea and vomiting were rarely complained of; the thirst was generally marked, though not insupportable; the appetite was entirely lost; respiration was somewhat accelerated, in proportion to the frequency of the pulse, and the heat of skin; cough was an exceptional symptom in this stage, and when present it was very slight and dry, and unconnected with any appreciable rale. Bleed ing at the nose occurred in four cases only, of the thirty-seven; at this period of the disease, the eye was clear but dull and heavy; thd cheek flushed, as in other febrile affections, acquiring at a latter moment the appearance common in typhus fever; the sense of hearing was slightly, if at all, obtuse.

The disease, when fully developed, was especially marked by increased oppression of the intellectual and sensorial functions. The mind became much more sluggish and dull; there was a more or less continued disposition to sleep during the day, frequently interrupted at night by delirium, generally of a non-violent character; muttering; the eyes suffused and void of animation; the cheeks covered with a dusky red flush. and the whole expression resembling that of a person very much intoxicated. The hearing was obtuse, sometimes very much so, accompanied generally by confused sounds, as of rumbling, or rolling, and the like. In one case only were there convulsions; but, with few exceptions, all had subsultus tendinum; in most this involuntary motion was confined to the rist and fingers; in some, the muscles of the fece, especially those about the mouth, were in a state of almost constant agitation. This latter symptom (agitation of the muscles of the face,) is properly regarded as a very unfavorable one; of the three patients who presented it in the most marked degree, two recovered. The convulsion, in the single case alluded to, occurred on the ninth day from his first complaining, and lasted but a very few moments; there was a slight twitching of the muscles of the face, both sides equally, the eyes became fixed, and the limbs perfectly rigid, this did not recur. But for the succeeding twenty-four hours, he presented some singular symptoms; when asked to protrude his tongue he replied that he could not; when asked any question, which would oblige him to converse, he replied thas he could not speak; he passed the entire twenty-four hours resting upon his left elbow, gently moving his body backwards and forwards, while in this position, and could not be induced to lie down. This patient recovered very speedily, being dressed and walking out on the sixteenth day from the commencement of his illness, on the tenth from his admission into the hospital.

The skin during this period became dry and hot; the calor mordax; the pulse increased in frequency, becoming at the same time more feeble, sometimes undulating, its frequency was variable; thus, of 15 cases in which it was noted, its greatest frequency was in each case respectively, 103: 98: 112: 80: 104: 128: 112: 100: 116: 105: 120: 124: 112: 140: 124. In two of the cases a remarkable diminution in the frequency of pulse occurred during convalescence; thus, in one patient whose pulse had been as high as 112, it fell during convalescence to 36 per minute; in another, from 80 to 42; in these two cases the pulse beat regularly throughout the illness; in a few others it became irregular during convalescence; and in others again some irregularity occurred only on the approach of death. The action of the heart was carefully noted: the impulse was found to be feeble in every case; both sounds were distinctly heard, without unnatural murmur. The respiration was more frequent than in health, but not more so than might have been expected considering the more frequent action of the heart; in only four out of thirty-seven cases, was there any abnormal sound audible in the respiratory movements. Iu one there was always mucous rale over the lower part of the right lung, from the root downwards, without bronchial respiration, or increased vocal resonance; the percussion was appreciably more dull than on the left side; the period of the disease in which this condition occurred could not be determined, from the want of any accurate knowledge as to the date of seizure. In two cases the same condition was first appreciable at the close of the second week. In the fourth instance,—a loose mucous rale existed about the root of the lungs, unaccompanied with dullness; this also appeared at the close of the second week.

In one case only was there distension of the abdomen; it was generally of about the same degree of fullness as is common in health; in many cases, however, it was decidedly retracted. In one case only was there any perceptible sensation of gurgling upon pressing in the right iliac region. Of 32 cases, 19 had no diarrhœa; of these latter 4 were costive. The remaining 13 patients had from four to eight passages daily; the evacuations being thin and yellowish, free from blood. The urine in all was small in quantity, of a reddish-brown color, emitting a decided ammoniacal odour upon standing a short time. In four patients there was retention of urine, requiring the use of the catheter. The tongue was dry, in many cases covered with a dark brown incrustation and fissured; the teeth and gums at the same time covered with sordes. The surface of the body was in every case, excepting one, abundantly covered with petechiæ; this exceptional case presented in a very marked degree all the other symptoms of typhus fever. The petechial spots were of a purplish-red color, becoming darker a few days after their appearance than at first; beneath the surface, and unaffected by pressure upon it; very numerous; generally circular in form, but sometimes oval, varying in size from a line or a line and a half to a fraction of a line in diameter. There was also another sort of eruption, consisting of spots of a much lighter hue than the first, sensibly elevated, in most instances; generally oval in shape, but sometimes circular, and again having no definite form; in size varying from a fraction of a line to two lines in diameter; partially, sometimes wholly, disappearing under pressure. Both of these varieties were diffused over the entire surface, excepting the face and forehead, but were most abundant on the abdomen; the paler variety was more numerous than the other. In six cases of which I had the opportunity of witnessing the whole course, from before the appearance of the eruption, this was first distinguishable on the eighth day in one case: on the sixth, in two; on the seventh, in one; on the fourth, in two. The two varieties appeared simultaneously, but the paler receded first; the precise period of its disappearance I am unable to state; it probably took place, however, in the early part of the third week. In addition to these smaller spots, there were in a few cases large purple blotches, superficial stains of the surface, in which the circulation was extremely languid. Of 32 cases, only three had sudamina. In one of these their time of appearance could not be determined; they were very numerous over the neck and upper part of the chest anteriorly:—in another instance they appeared in the early part of the third week; they were not abundant, and were confined to the breast:—in the third, they appeared for the first time on the eleventh day upon the breast; had all vanished in the course of twenty-four hours, and again reappeared on the fourteenth day, very numerous and very large, and were diffused very generally over the trunk and extremities, being most abundant, however, over the abdomen. The peculiar odour exhaled from the body, noticed as existing in cases of typhus fever, was appreciable in the first period of the disease in most of the patients, and to a greater degree in the fully developed affection. At no time, however, and in no instance was it so very heavy and offensive, as it has been found to be in many epidemics of typhus fever.

There was more or less bleeding from the nose in 11 patients out of 32, during this second period; in one of these there had been previous bleedings; it recurred on the eighth day of the disease. The precise date of the bleeding could be ascertained only in one other instance; in this it occurred on the ninth day. The hemorrhage in all, excepting one instance, was very slight, and should rather be called an oozing of a thin pale-red fluid; in one case, however, it was very abundant, and was arrested with great difficulty, and only after the individual had fainted from loss of blood: this occurred before the ship arrived at the quarantine ground; the patient eventually recovered. In a few cases a similar oozing appeared to take place from the gums.

I can only state the period at which the change in the degree of illness occurred, in five patients, constituting what has been termed herein, the second period; the other patients came under my observation after this stage was fully developed. Considering this stage as characterized particularly by obtuseness of the cerebral functions it was developed in one case on the 8th day; in one on the 9th; in two on the 7th; in one on the 5th.

The ages of the patients were as follows: under 6 years, 3; from 6 to 20, 5; from 20 to 30, 17; from 30 to 40, 7; from 40 to 50, 5. The duration of the disease, from the time of sickening until the patient was able to walk about, free from ailment, was, as nearly as could be ascertained, in fourteen cases, as follows: 13 days in 3 cases; 14 in 1; 15 in 2; 16 in 3; 18 in 1; 19 in 1; 20 in 1; 21 in 1; 22 in 1. From the time that the patient became entirely convalescent, free from fever, &c., until he was sufficiently strong to be dressed, and to walk about the wards, an interval of only 3 days elapsed in 9 cases; of 4 days in 3; of 6 days in 2. Death occurred in one case on the 7th day of the illness; in three on the 10th; in one on the 17th.

The degree of emaciation was very moderate in all the patients who recovered; the digestive powers were speedily restored to their full force, and the natural embonpoint and strength quickly regained.

The appearances after death in four subjects which were carefully examined, were the following:

*Externally.*—Moderate emaciation; rigidity of limbs; large purple patches at the flexures of the limbs, and on the inferior parts of the surface; no sudamina; in three the deep purplish-scarlet circular pntechial spots remained more or less numerous, and diffused over the trunk and limbs, the lighter-colored spots having disappeared; the abdomen was retracted in all. The eyes were sunken, and the features pinched.

*Contents of Cranium.*—Cerebrum of excellent consistance. Cerebellum very slightly softened in three of the subjects. The venous sinuses were moderately full of a dark-colored blood, without coagulum; the ventricles contained, in each case, from ʒj to ʒij of serum, slightly stained; no injection of membranes, and no adhesion of them to the surface of the brain. A freshly cut surface of the brain exhibited not more than the usual number of bloody points.

The *Heart* in all had a somewhat flabby, soft feel, though the consistence of the walls was not appreciably diminished. The valves in all were healthy; the lining membrane of the cavities of the large trunks was stained; the blood was dark-colored and fluid, one only containing a coagulum, which was small, very soft, and pale. In all, the size of the heart was normal. The pericardial sac contained a few drachms of slightly-reddish serum, free from flocculi; the lining membrane unaltered.

The *Lungs*, in two instances, were perfectly healthy, excepting that some degree of congestion existed at the inferior posterior part; in one of these, a few delicate, pale bands of lymph were stretched across the right pleural cavity, evidently of old formation; in each of these cases, the pleural cavities contained between ℥ss and ℥j of stained serum, without flocculi. The bronchial membrane was stained corrospondingly with the tissue proper of the lungs, this stain was uniform, dark-colored, without abrasion or softening of the membrane. Death took place in these two instances on the tenth, and on the seventh day. In the other two instances, the right lung in each was slightly softened in the lower lobe, (which, however, contained air,) and deeply stained. The cavity of the right pleura was, in each instance, traversed by bands of lymph, pale in one, in the other stained, in both very firm; ℥j of stained serous fluid existed in each; in one of these latter instances the liver was firmly and closely attached to the diaphragm, by false membranes of old formation; in this case, death occurred on the seventeenth day. The bronchial glands were unaffected.

The *Liver* was not appreciably altered in any case.

The *Spleen* seemed of good consistence in all—tearing with a granular surface, and resisting pressure about as well as the spleen usually dose. Its size varied;—in one instance, it measured 3 by 3½ inches; in one, the dimensions were 2½ by 4 inches; in a third, 3 by 5; in a fourth, 4 by 7 inches. The color of the organ was bluish, with a perceptible tinge of green.

The *Kidneys* felt soft and flabby, but their consistence did not appear to be really diminished.

The *Bladder* offered no appearance of disease.

The *Stomach*, in three of the four subjects, was pale; in one, it was deeply stained at its greater curvature. In none was there any softening or abrasion of the mucous membrane; in two this was covered with a thick, viscid semi-fluid matter of a yellowish color. The organ was at a medium degree of distension in all.

The *Intestinal canal* in one instance was uniformly stained from the lower extremity of the jejunum to the transverse colon; there was no softeoing of the mucous membrane in this case, nor abrasion, nor inflammatory injection, simply, a dark-colored stain; the larger veins were turgid. The isolated follicles were visible, scattered here and there throughout the length of the small intestine, and less numerously in the upper part of the colon. The agmination follicles were very apparent in the lower half particularly of the ileum; the last three feet of the small intestine contained five of these patches of Peyer. The mucous membrane covering them was stained, as elsewhere, and the tissue of the glands themselves apparently slightly reddened also, the lining membrane of the canal was not very sensibly elevated at these points, nor abraded at all. Some of the mesenteric glands, near the lower extremity of the ileum, were enlarged and injected, of a reddish-brown color; most were very small; a few measured from ½ to ¾ of an inch in the long diameter. No softening in any instance.

In the other three subjects, the mucous membrane of the intestinal canal was pale. No turgescence of the large veins. In two, the glands of Peyer could not be seen at all with the naked eye; in in the third subject, they were numerous, their outlines well marked and the bluish dotted appearance within; no thickening, abrasion or discoloration of the mucous membrane covering them. In one the isolated follicles were not perceptible; in the other two, they were sufficiently numerous. In these three instances, the mesenteric glands were pale, and the largest scarcely the size of a pea. The large intestine exhibited no evidence of inflammation or softening, or any disease.

The serous membrane of the abdomen presented no departure from its ordinary healthy condition, excepting in the single instance already mentioned, in which the opposed surfaces of the liver and diaphragm were adherent.

The treatment consisted in the administration of tonics and stimulants, with the use of calomel in very small doses, combined with ipecac. or Dover's powder, or opium alone. The preparations of Peruvian bark, and chiefly sulphate of quinia, were the tonics made use of. Punch, carbonate of ammonia, brandy, &c., were the stimulant remedies most resorted to. As local applications, I found frequent cold sponging of the surface of great service, as were likewise blisters in some cases; also dry and wet cups, and stimulating lotions. The diet consisted of farinaceous articles, broth, essence of beef, and, upon convalescence, a mixed animal, and vegetable regimen.—*American Journal of Med. Sciences.*

